# Study of *rs7759938*, *rs314280*, and *rs314276* Polymorphisms of *LIN28B* in Relation to Age at Menarche in Girls of Greek Descent

**DOI:** 10.3390/children11080912

**Published:** 2024-07-29

**Authors:** Vasiliki Rengina Tsinopoulou, Flora Bacopoulou, Liana Fidani, Dimitrios Dimitriadis, Spyridon Gerou, Athanasios Christoforidis

**Affiliations:** 1 2nd Department of Pediatrics, School of Medicine, Faculty of Health Sciences, Aristotle University of Thessaloniki, University General Hospital AHEPA, 54636 Thessaloniki, Greece; 2 Center for Adolescent Medicine and UNESCO Chair in Adolescent Health Care, 1st Department of Pediatrics, School of Medicine, National and Kapodistrian University of Athens, Aghia Sophia Children’s Hospital, 11527 Athens, Greece; 3 Laboratory of Genetics, School of Medicine, Faculty of Health Sciences, Aristotle University of Thessaloniki, 54636 Thessaloniki, Greece; 4 School of Economics, Aristotle University of Thessaloniki, 54124 Thessaloniki, Greece; 5 Analysis Biopathological Diagnostic Research Laboratories, 54623 Thessaloniki, Greece; 6 1st Department of Pediatrics, School of Medicine, Faculty of Health Sciences, Aristotle University of Thessaloniki, Ippokratio General Hospital, 54636 Thessaloniki, Greece

**Keywords:** *LIN28B*, menarcheal age, single-nucleotide polymorphisms

## Abstract

Background: Single-nucleotide polymorphisms in *LIN28B*, critical regulators of female growth and puberty, have been linked to age at menarche. Methods: We assessed the association of *rs7759938*, *rs314280*, and *rs314276* with menarcheal age in girls of Greek descent. We reviewed the records of 248 girls who had their first menstruation before 18 years and who attended the Greek Departments of Pediatric Endocrinology from January 2021 to July 2023. Genotyping was performed by standard DNA-based methods. Association analyses involved both parametric and non-parametric tests. Results: The average age of breast and pubic hair development was 9.95 years, and the age at menarche was 11.55 years. Menarche occurred ≤11 years (mean 10.24 years) in 108 girls (43.5%) and >11 years (mean 12.55 years) in 140 (56.5%). The girls’ menarcheal age correlated significantly with that of their mothers (average 12.1 years, *p*-value < 0.0001, Spearman’s r 0.350). The dominant *rs7759938(TT)* genotype was the most common (55.2%), followed by the dominant *rs314276(CC)* (53.2%) and dominant *rs314280(TT)* (14.5%) genotypes. Conclusions: There was no association between age at menarche and any of the polymorphism genotypes/alleles or between genotypes/alleles and birth weight, gestational week, mode of delivery, and maternal age at menarche. Future large sample studies are warranted to confirm these results.

## 1. Introduction

Menarche, the first menstrual period, signals the start of female reproductive capability and is a significant event in puberty. Menarche typically occurs between the ages of 10 and 16 years, with an average onset of 12.4 years [1]. The timing of menarche dictates subsequent stages of pubertal development and has substantial long-term health implications, such as behavioral and psychosocial issues during adolescence [2], as well as fertility difficulties and increased risk of certain diseases in adulthood. Late menarche (after 16 years) has been linked to infertility [3] and has been shown to increase the risk of osteopenia and osteoporotic fractures [4]. Conversely, early menarche (before 12 years) is suggested to increase the risk of breast cancer [5], different gynecological cancers [6,7], and cardiovascular disease [8]. Additionally, early menarche is linked to high blood pressure, glucose intolerance, a higher risk of metabolic syndrome, increased body mass index (BMI) [9,10], and high mortality rates [8]. 

Studies involving families and twins have indicated a genetic component to the age at menarche and pubertal onset, explaining up to 80% of the variance in pubertal onset [11,12,13]. Genome-wide association studies (GWASs) have revealed several genetic loci linked to age at menarche [14,15], with *LIN28B* standing out as a critical regulator of female growth and puberty [16,17,18,19]. *LIN28B*, a human equivalent of *Lin28* in C. elegans, is an RNA-binding protein that governs the degradation of let-7 microRNAs. GWASs have identified the connection between polymorphism in or around the *LIN28B* gene and the age at menarche across various population groups globally [15,16,17,18,19,20,21]. 

The aim of this retrospective study was to examine the association of three *LIN28B* single-nucleotide polymorphisms, *rs7759938*, *rs314280*, and *rs314276*, with age at menarche in girls of Greek descent.

## 2. Materials and Methods

### 2.1. Study Population

This study enrolled girls of Greek descent who attended two Departments of Pediatric Endocrinology from January 2021 to January 2023. Participants were included if their menstruation had begun until the age of 18 years and if they and their parents/guardians provided consent for participation in this study. Participants were excluded if they were older than 18 years old, were of any descent other than Greek, and had any chronic conditions that could influence the timing of pubertal stages. Moreover, those with extreme weight, height, or body mass index (BMI) above the 95th percentile or below the 3rd percentile for their age and race as determined by the percentiles for growth and development of Centers for Disease Control and Prevention, as well as those who refused to participate in this study, were also excluded. 

The enrolled participants were divided into two study groups based on their age at menarche: 11 years or younger (Group 1) and older than 11 years (Group 2). 

All enrolled participants and their parents/guardians provided written informed consent to participate in this study. This study was also conducted according to the principles of the Declaration of Helsinki and was approved by the Bioethics Committee of the School of Medicine, Faculty of Health Sciences of the Aristotle University of Thessaloniki, Greece (Protocol No. 3.297, 3/22 December 2020).

### 2.2. Data Collection and Analysis

The exact age at menarche was recorded. Age was first converted to months and then divided by 12 to yield the decimal age in years (e.g., 53 months/12 = 4.41 years). Birth weight, birth length, and head circumference at birth were extracted from the participants’ medical records and placed in the corresponding percentiles of growth according to Fenton’s gestational age, i.e., small for gestational age (SGA), of normal weight for gestational age [appropriate for gestational age (AGA)], and large for gestational age (LGA) [22]. Data regarding the gestational age (premature or full-term pregnancy), the mode of delivery (cesarean section or vaginal delivery), and the order of birth among siblings in the family (first born, second born, etc.) were also recorded. In addition, the baseline values of gonadotropins (LH and FSH) and estradiol, as well as the results of thyroid function assessments (free thyroxine (fT4), thyroid-stimulating hormone (TSH), and thyroid autoantibodies such as thyroid peroxidase antibodies (anti-TPO) and anti-thyroid microsomal antibodies (anti-Tg)), were recorded from the participants’ medical histories. The age at menarche of each participant’s mother was documented from the family history. 

Finally, at the time of recruitment, the age, weight (in kilos, kg), height (in centimeters, cm), and BMI (in kg/m^2^) were recorded, and the corresponding percentile positions in the growth curves of the CDC. 

### 2.3. DNA Isolation and Genotyping 

Two samples of total peripheral blood, 2 mL each, were drawn from all participants in vials coated with anticoagulant ethylenediaminetetraacetic acid (EDTA) and were stored at −80 °C until use. 

Total DNA was isolated using the HigherPurityTM Blood DNA Extraction Kit (Canvax Biotech, Valladolid, Spain).

Detection of *rs7759938* and *rs314280* polymorphisms was performed using real-time polymerase chain reaction (RT-PCR) with gene-specific primers, as well as the Taqman SNP Genotyping Assays reagent system, according to the manufacturer’s instructions (Thermo Fisher Scientific, Waltham, MA, USA). Melting curve analysis was performed to detect rs314276 by using gene-specific primers and the LightCycler^®^ FastStart DNA Master HybProbe reagent system (F. Hoffmann-La Roche, Basel, Switzerland). 

### 2.4. Statistical Analysis

Statistical analysis was performed using the IBM SPSS software (version 27); the significance level was set at 5% in all tests performed. Normal distribution of the age at menarche was examined by Kolmogorov–Smirnov and Shapiro–Wilk tests. Since the null hypothesis of normal distribution of these tests was rejected, the difference in the age at menarche among the three possible genotypes of each polymorphism was assessed using the non-parametric Kruskal–Wallis test. The non-parametric Mann–Whitney U test with Bonferroni error correction was used for multiple comparisons. The χ^2^ test of independence was used to study possible associations between genotype and/or allele and the age at menarche (4 groups: <10, 10–11, >11 or ≤15, and >15 years). 

#### 2.4.1. Sample Size Calculation 

The sample size for this study was determined from a previous pilot study in which test power $\left(1-b\right)$ was set equal to 0.95 and the level of significance equal to a=0.05. The data were analyzed into a z−test family procedure of (difference between two independent proportions) with G*power 3.1 for Windows software. The sample ratio was set to 1.3 and the optimal sample size was calculated up to 248 statistical units. Specifically, 108 statistical units for Group 1 (age ≤11 years) and 140 statistical units for Group 2 (age >11 years).

#### 2.4.2. Association Analyses

Logistic regression (Z test) was performed to examine the association between genotype and the age at menarche. Associations between age groups and genotype/allele patterns were estimated by odds ratios (ORs) and 95% confidence intervals (CIs). Different genetic models were recruited to examine the abovementioned associations. All models that were used are described below:

- The dominant model, in which the homozygous genotype was used as a reference (*TT* for *rs7759938* and *rs314280*, *CC* for *rs314276*) to investigate the prevalence of mutant-allele-associated genotypes (*CC* and *TC* for *rs7759938* and *rs314280*, *TT* for *rs314276*) within age groups.
- The recessive model, in which wild-type-allele-associated genotypes (*TC* and *TT* for *rs7759938* and *rs314280*, *TC* and *CC* for *rs314276*) were used as a reference to investigate the prevalence of the mutant homozygous genotype (*CC* for *rs7759938* and *rs314280*, *TT* for *rs314276*) within age groups.
- The allelic model, in which the wild-type allele (*T* for *rs7759938* and *rs314280*, *C* for *rs314276*) was used as a reference to investigate the prevalence of the mutant allele (*C* for *rs7759938* and *rs314280*, *T* for *rs314276*) within age groups.

In the first step of the analysis, the “Dominant” (major allele homozygotes vs. heterozygotes + minor allele homozygotes) and “Recessive” (major allele homozygotes + heterozygotes vs. minor allele homozygotes) models were created for each polymorphism, and their correlations with age at menarche ≤ 11 and >11 years were tested. In the second step, allelic models were created, where each allele was examined separately and not as part of the genotype, and its association with the age at menarche was examined again. The association of genotype with the participants’ perinatal history (birth weight for gestation age (AGA, LGA, SGA), type of delivery (C-section, normal birth), and gestation week) was assessed by chi-squared test for qualitative variables and by Kruskal–Wallis H or Mann–Whitney U test for quantitative variables, followed by Bonferroni’s correction for multiple comparisons. The correlation between the age at menarche of participants and that of their mothers was examined using Spearman correlation. Kruskal–Wallis H was used to assess the association between the participants’ genotype and their mother’s age at menarche. Finally, to assess the correlation between body weight, age at menarche, and *LIN28B* polymorphisms, overweight participants were selected, and the association between genotype and their age at menarche was examined by Kruskal–Wallis H, followed by the Mann–Whitney U test and Bonferroni’s correction for multiple comparisons in case of significant results.

## 3. Results

### 3.1. Subject Characteristics and Genotypes 

A total of 300 Greek girls under 18 years who had experienced menarche were recruited. Due to exclusion criteria, 52 girls were excluded, resulting in a final sample of 248 girls for the analysis. Reasons for exclusion included celiac disease (*n* = 2), thyroid disease (*n =* 7), type 1 diabetes mellitus (*n* = 3), use of puberty blockers (*n* = 10), obesity (*n* = 11), height above 95th percentile for age (*n* = 6), social reasons (*n* = 5), and personal refusal to participate (*n* = 8).

The characteristics of all participants, along with their perinatal history and family history, are shown in Table 1. The average age of breast development was 9.95 years (SD = 1.45), the average age of pubic hair development was 9.93 years (SD = 1.46), and the average age at menarche was 11.55 years (SD = 1.54). The average maternal age at menarche was 12.11 years (SD = 1.99) and correlated significantly with the daughters’ age at menarche (*p*-value < 0.0001, Spearman’s r 0.350). All girls in this study were euthyroid with negative thyroid autoantibodies and hormone levels compatible with the Tanner stage and cycle phase in which they were measured.

Overall, 108 out of 248 girls (43.5%) experienced menarche before or at the age of 11 years (Group 1), and 140 out of 248 (56.5%) after 11 years (Group 2). Group 1 had a mean age at menarche of 10.24 years (SD = 0.74) and, at the time of recruitment, a mean weight of 53.19 kg (SD = 11.64), a mean height of 156.03 cm (SD = 10.71), and a mean body mass index (BMI) of 21.57 kg/m^2^ (SD = 3.08) (Table 1). Group 2 had a mean age at menarche of 12.55 years (SD = 1.21) and, during recruitment, a mean weight of 56.11 kg (SD = 11.26), a mean height of 160.81 cm (SD = 7.96), and a mean BMI of 21.57 kg/m^2^ (SD = 3.26) (Table 1).

#### 3.1.1. Subjects’ Perinatal History

The mean gestational duration, birth length, and birth weight in Group 1 were 38.17 weeks (SD = 1.59), 49.28 cm (SD = 3.87), and 3.130 kg (SD = 1.59), respectively (Table 1). Based upon birth weight, 75.9% were AGA, 13.9% were SGA, and 10.2% were LGA in Group 1; similar percentages were observed in Group 2, where 74.3% were AGA, 13.6% SGA, and 12.1% LGA (Table 1). The mean gestational duration in Group 2 was 38.40 weeks (SD = 1.46), the mean birth weight was 3.194 kg (SD = 0.45), and the mean birth length was 49.52 cm (SD = 2.36). 

#### 3.1.2. Genotypes 

The frequencies of genotypes for all *LIN28B* polymorphisms are shown in Table 2. For *rs7759938*, the homozygous (*TT*) genotype was the most common, found in approximately 55% of all participating girls, while heterozygosity (*TC)* and homozygosity (*CC)* for the recessive allele were detected in 44.5% of the girls (Table 2). In *rs314280,* the dominant [*TT*] genotype was found only in 14.5% of all girls, with the heterozygous (*CT*) and homozygous (*CC*) genotypes being the most common, found in 85.5% of the girls. Finally, the *rs314276* dominant (*CC*) genotype was detected in 53.2% of the girls. 

In relation to the age at menarche, no significant differences were detected in the distribution of the dominant models of all polymorphisms (*rs7759938(TT)*: z-score: 0.029, *p*-value: 0.865, 95% CI: 0.578–1,585; *rs314280(TT)*: z-score: 0.948, *p*-value: 0.330, 95% CI: 0.335–1.447; *rs314276(CC)*: z-score: 0.815, *p*-value: 0.367, 95% CI: 0.762–2.089), as shown in Table 3. 

Accordingly, no statistically significant differences were observed in the distribution of the dominant allele among polymorphisms (*rs7759938*(*T*): z-score: 0.061, *p*-value: 0.806, 95% CI: 0.639–1.417; *rs314280(T):* z-score: 2.395, *p*-value: 0.122, 95% CI: 0.520–1.080; *rs314276(C)* z-score: 0.261, *p*-value: 0.610, 95% CI: 0.607–1.341) as shown in Table 4. 

The frequency of homozygosity of recessive genotypes was also not significantly different between groups, according to age at menarche (Table 3). Testing of the association of age at menarche considering four age groups based upon menarcheal age (<10 years, 10–11 years, 11–15 years, and >15 years) with the polymorphisms also failed to reveal any significant result (Table 5).

Following, we examined the association of different participants’ characteristics with their genotypes. The relationship between birth weight for gestational age (SGA, AGA, and LGA) and genotype did not emerge as statistically significant for any of the three genotypes of polymorphisms *rs7759938* (*p*-value: 0.691), *rs314280* (*p*-value: 0.278), and *rs314276* (*p*-value: 0.436) (Table 6). Corresponding findings emerged from assessing the relationship with the dominant alleles of the point polymorphisms (*rs7759938*: *p*-value: 0.679; *rs314280*: *p*-value: 0.315; *rs314276*: *p*-value: 0.139) (Table 6). 

No significant association was detected between menarcheal age and polymorphisms in girls who were overweight at enrolment (*rs7759938*: *p*-value: 0.543; *rs314280*: *p*-value: 0.455; *rs314276*: *p*-value: 0.994), and there was also no correlation between the type of delivery and the genotypes (*rs7759938*: *p*-value: 0.494; *rs314280*: *p*-value: 0.415; *rs314276*: *p*-value: 0.677) or the alleles of the polymorphisms (*rs7759938*: *p*-value: 0.229; *rs314280*: *p*-value: 0.187; *rs314276*: *p*-value: 0.419) (Table 6). 

Finally, the relationship between the gestational week and the dominant allele in the genotype was also not statistically significant for any of the three polymorphisms (*rs7759938(T): p*-value: 0.484; *rs314280(T)*: *p*-value: 0.946; *rs314276(C*) *p*-value: 0.458) (Table 6). 

The only relationship that emerged as statistically significant from this study was that between girls’ menarcheal age and their mothers’ menarcheal age (*p*-value < 0.0001). However, further analysis of the girls’ genotype with their mothers’ age at menarche did not yield significant results for any of the three polymorphisms *rs7759938* (*p*-value: 0.421), *rs314280* (*p*-value: 0.886), and *rs314276* (*p*-value: 0.730) (Table 5).

## 4. Discussion

This study analyzed the genotypes and alleles of the *rs7759938, rs314280*, and *rs314276* single-nucleotide polymorphisms of *LIN28B* in 248 adolescent girls of Greek descent, divided into two groups based on their age at menarche (before and after 11 years). Statistical analyses revealed no association between the participants’ age at menarche and any of the three polymorphisms, either at the genotype or allele level. Testing the association of these polymorphisms with specific parameters from the girls’ perinatal history also yielded no significant results. Overall, the only significant relationship arising from this study was between the age at menarche of the girls and that of their mothers. 

Previous studies have highlighted the significance of *LIN28B* in puberty. In Chinese girls, Li et al. [23] found that the recessive *C* allele of the *rs7759938* polymorphism is linked to early and accelerated puberty, while Hu et al. [21] indicated that the *CC* genotype of *rs7759938* and the *AA* genotype of *rs314280* are linked to a lower risk of idiopathic central precocious puberty (CPP) and that the *TT* or *TC* genotype of *rs7759938* and the *GG* or *GA* genotype of *rs314280* increase idiopathic CPP susceptibility. In US Hispanic girls, Chen et al. [24] showed that the *C* allele of *rs314276* in *LIN28B* renders carriers more susceptible to CPP. *LIN28B* was also associated with earlier development of puberty-related body characteristics, such as breast development and pubic hair, a more rapid height growth spurt during puberty in girls, and shorter adult height in women [17], as well as with higher weight and BMI [25]. 

In this study, which included girls of Greek origin, there was no association of any genotype or allele of the *rs7759928*, *rs314280*, and *rs314276* polymorphism of *LIN28B* with the girls’ weight at enrolment or with their birth weight. Similarly, in a large Finnish cohort, the *rs7759938* variant was significantly linked to adult height but showed no significant association with weight, BMI, or waist and hip circumference [26]. In the study by Ong et al. [25], longitudinal analyses revealed age-dependent associations between *rs314276* genotype and BMI and body weight in women. Specifically, the *rs314276 C* allele was shown to be associated with greater weight and BMI during adolescence and early adult life in women, but its effects were less pronounced in women aged 30–40 years and older [25]. A positive association with body weight and BMI was found for polymorphism *rs314276* in the Finish population [26], indicating that not all *LIN28B* variants are associated with adult body characteristics. Variances in lifestyle factors among different populations may also influence these results. 

In this study, there was no correlation between the examined *LIN28B* polymorphisms and the participants’ age at menarche, a hallmark of puberty development in girls. In one of the initial GWAS identifying *LIN28B* polymorphisms in relation to the age at menarche conducted in 2009, Ong et al. showed *rs314276* as a polymorphism conferring 0.12 years earlier menarche [17]. In contrast, in a Japanese population-based study conducted a few years later, including 15000 women, *rs314276* was found to be associated with later menarche by 0.085 years [20]. Opposite to our study results, the *rs314280* polymorphism has been consistently shown to confer late menarche in different population cohorts, including European American, Asian, North Hawaiian, Japanese, Taiwanese (Han Chinese descent), and Finnish [14,16,19,20,27,28,29], whereas the *rs7759938* polymorphism was associated with earlier menarche in Filipino and Japanese women but with late menarche in women of Chinese and European descent [15,18,20,28,30,31,32]. The frequency of different polymorphisms in distinct cohorts, as well as the linkage disequilibrium between polymorphisms [33] in a given cohort, may explain the discrepancies among studies. 

The significant relationship between the mothers’ age at menarche and the girls’ age at menarche found in this study is consistent with the previous results. Indeed, the maternal age at menarche was found to be a strong predictor of the age at menarche in daughters [34,35,36]. However, no significant relationship was identified with the daughters’ genotypes of polymorphisms studied. Moreover, no relationship was established between any of the three polymorphisms of *LIN28B* and various perinatal parameters such as duration of gestation, birth weight for gestational age, and mode of delivery. In a study of girls recruited in rural Bangladesh, birth size was not a significant predictor of the age at menarche [37], whereas in another study, early menarche (before or at 10 years or 11 years) was associated with having low birth weight, having had a teenage mother, and being firstborn [38]. Since we did not find an association between the girls’ genotypes in *LIN28B* and age at menarche, it may be reasonable that we also did not find an association of the polymorphisms with the above-mentioned perinatal factors.

This study should be interpreted considering some limitations. First, the data collection was limited to the available medical records of the participants. Second, the sample size, even though sufficient for the study objective, was relatively small compared with that of other large cohort studies; in the association analyses, we did not account for confounding factors that could have influenced the results. Finally, we did not perform multifactorial analyses, which could have revealed interactions between *LIN28B* polymorphisms, the age at menarche, and other participants’ characteristics. Therefore, future large-cohort prospective studies are warranted to verify our results. 

In conclusion, this study, even though it was the first to examine *LIN28B* polymorphisms in the Greek population, could not confirm a relationship between menarcheal age and the *LIN28B* polymorphisms *rs7759938*, *rs314280*, and *rs314276* or between these polymorphisms and other cohort traits in girls of Greek descent. Further studies are needed to confirm these findings in this population. 

## Figures and Tables

**Table 1:** Characteristics of participants.

	Overall (*n* = 248)		Menarcheal Age ≤11 Years (*n* = 108)		Menarcheal Age >11 Years (*n* = 140)	
Parameter	Mean ± SD *	*n* (%)	Mean ± SD	*n* (%)	Mean ± SD	*n* (%)
Age at menarche (years)	11.55 ± 1.55		10.24 ± 0.74		12.55 ± 1.21	
Age at thelarche (years)	9.95 ± 1.45					
Age at adrenarche (years)	9.93 ± 1.46					
At enrolment						
Age (years)	13.7 ± 2.28		13.0 ± 2.57		14.26 ± 1.84	
Weight (kg)	54.84 ± 11.50		53.19 ± 11.64		56.11 ± 11.26	
Height (cm)	158.73 ± 9.54		156.03 ± 10.71		160.81 ± 7.96	
BMI * (kg/m^2^)	21.57 ± 3.19		21.57 ± 3.08		21.57 ± 3.26	
Residence						
Urban				70 (64.81)		80 (57.14)
Rural				38 (35.10)		60 (42.85)
Perinatal history data						
Gestational week	38.30 ± 1.52		38.17 ± 1.59		38.40 ± 1.46	
Birth weight (kg)						
SGA *		34 (13.70)		15 (13.90)		19 (13.60)
AGA		185 (74.6)		82 (75.9)		104 (74.30)
LGA		28 (11.3)		11 (10.20)		17 (12.10)
Birth length (cm)	49.46 ± 2.85		49.28 ± 3.87		49.59 ± 2.36	
Mode of delivery						
Vaginal delivery		129 (52)		61 (56.50)		68 (48.60)
Cesarian section		119 (48)		47 (43.50)		72 (51.40)
Order in family						
1st child		144 (58.10)		63 (58.30)		81 (57.90)
2nd child		70 (28.20)		34 (31.50)		36 (25.70)
3rd child		27 (10.90)		8 (7.40)		19 (13.60)
4th child		7 (2.80)		3 (2.80)		4 (2.90)
Family history						
Mothers AAM *	12.10 ± 1.99		11.64 ± 1.86		12.47 ± 2.03	
Mothers’ height (cm)	161.91 ± 18.95		161.42 ± 16.89		162.28 ± 20.45	

* SD, standard deviation; BMI, body mass index; SGA, small for gestational age; AGA, appropriate for gestational age; LGA, large for gestational age; AAM, age at menarche.

**Table 2:** Genotype frequencies of polymorphisms *rs7759938*, *rs314280*, and *rs314276* in *LIN28B*.

	Overall (*n* = 248)	Menarcheal Age ≤11 Years (*n* = 108)	Menarcheal Age >11 Years (*n* = 140)
Polymorphism	*n* (%)	*n* (%)	*n* (%)
Genotype			
*rs7759938*			
*TT*	137 (55.20)	59 (54.60)	78 (55.70)
*CC*	24 (9.70)	11 (10.20)	13 (9.30)
*TC*	87 (35.10)	38 (35.20)	49 (35.0)
*rs314280*			
*TT*	36 (14.50)	13 (12.00)	23 (16.40)
*CC*	88 (35.50)	44 (40.70)	44 (31.40)
*TC*	124 (85.50)	51 (88.00)	117 (83.60)
*rs314276*			
*CC*	132 (53.20)	61 (56.50)	71 (50.7)
*TT*	23 (9.30)	11 (10.20)	12 (8.60)
*CT*	116 (46.80)	47 (43.50)	69 (49.30)

**Table 3:** Association of dominant and recessive models of polymorphisms *rs7759938, rs314280*, and *rs314276* in *LIN28B* with age at menarche.

	Overall (*n* = 248)	Menarcheal Age ≤11 Years (*n* = 108)	Menarcheal Age >11 Years (*n* = 140)			
Polymorphism	*n* (%)	*n* (%)	*n* (%)	z-Score	*p*-Value	95% CI
Genotype						
Dominant model						
*rs7759938*				0.029	0.865	0.578–1.585
*TT*	137	59	78			
*TC/CC*	111	49	62			
*rs314280*				0.948	0.330	0.335–1.447
*TT*	36	13	23			
*TC/CC*	212	95	117			
*rs314276*				0.815	0.367	0.762–2.089
*CC*	132	61	71			
*TC/TT*	116	47	69			
Recessive model						
*rs7759938*				0.056	0.812	0.476–2.580
*CC*	24	11	13			
*CT/TT*	224	97	127			
*rs314280*				2.309	0.129	0.888–2.533
*CC*	88	44	44			
*CT/TT*	160	64	96			
*rs314276*				0.189	0.664	0.512–2.857
*TT*	23	11	12			
*TC/CC*	225	97	128			

**Table 4:** Association of alleles of polymorphisms *rs7759938*, *rs314280*, and *rs314276* in *LIN28B* with age at menarche.

	Overall (*n* = 496)	Menarcheal Age ≤11 Years (*n* = 216)	Menarcheal Age >11 Years (*n* = 280)			
Polymorphism	*n* (%)	*n* (%)	*n* (%)	z-Score	*p*-Value	95% CI
Allele						
*rs7759938*				0.061	0.806	0.639–1.417
*T*	361 (72.80)	156 (72.20)	205 (73.20)			
*C*	135 (27.20)	60 (27.80)	75 (26.80)			
*rs314280*				2.395	0.122	0.520–1.080
*T*	196 (39.50)	77 (35.60)	119 (42.50)			
*C*	300 (60.5)	139 (64.40)	161 (57.50)			
*rs314276*				0.261	0.610	0.607–1.341
*C*	357 (72.00)	158 (73.10)	199 (71.10)			
*T*	139 (28.00)	58 (26.90)	81 (28.90)			

**Table 5:** Association of genotypes and alleles of polymorphisms *rs7759938*, *rs314280*, and *rs314276* in *LIN28B* with age at menarche in the 4 groups.

Polymorphism	<10 Years	10–11 Years	>11 and ≤15 Years	>15 Years	
Genotype Allele	*n* (%)	*n* (%)	*n* (%)	*n* (%)	*p*-Value
*rs7759938*					
*TT*	14 (42.4)	45 (60.0)	70 (54.3)	8 (72.7)	0.562
*TC*	14 (42.4)	24 (32.0)	47 (36.4)	2 (18.2)	
*CC*	5 (15.2)	6 (8.0)	12 (9.3)	1 (9.1)	
*T*	42 (63.6)	114 (76.0)	187 (72.5)	18 (81.8)	0.213
*C*	24 (36.4)	36 (24.0)	71 (27.5)	4 (18.2)	
*rs314280*					
*TT*	6 (18.2)	7 (9.3)	21 (16.3)	2 (18.2)	0.586
*TC*	16 (48.5)	35 (46.7)	67 (51.9)	6 (54.5)	
*CC*	11 (33.3)	33 (44.0)	41 (31.8)	3 (27.3)	
*T*	28 (42.4)	49 (32.7)	107 (41.8)	10 (45.5)	0.255
*C*	38 (57.6)	101 (67.3)	149 (58.2)	12 (54.5)	
*rs314276*					
*TT*	18 (54.5)	43 (57.3)	63 (48.8)	8 (72.7)	0.474
*TC*	10 (30.3)	26 (34.7)	55 (42.6)	2 (18.2)	
*CC*	5 (15.2)	6 (8.0)	11 (8.5)	1 (9.1)	
*T*	20 (30.3)	38 (25.3)	77 (29.8)	4 (18.2)	0.534
*C*	46 (69.7)	112 (74.7)	181 (70.2)	18 (81.8)	

**Table 6:** Association of different participants’ characteristics with the frequency of genotypes and alleles of polymorphisms *rs7759938*, rs314280, and *rs314276* in *LIN28B*.

	*p*-Value		
Parameter	*rs7759938*	*rs314280*	*rs314276*
Gestation duration (weeks)			
allele	0.484	0.946	0.458
Birth weight (SGA, AGA, LGA)			
genotype	0.691	0.278	0.436
allele	0.679	0.315	0.139
Age at menarche for overweight girls (years)			
genotype	0.543	0.455	0.994
Type of delivery (C-section, natural)			
genotype	0.494	0.415	0.677
allele	0.229	0.187	0.419
Mother’s age at menarche (years)			
genotype	0.421	0.886	0.730

## Data Availability

The raw data supporting the conclusions of this article will be made available by the corresponding author on request.
